# *KMT2C*, a histone methyltransferase, is mutated in a family segregating non-syndromic primary failure of tooth eruption

**DOI:** 10.1038/s41598-019-52935-7

**Published:** 2019-11-11

**Authors:** Ali A. Assiry, Alia M. Albalawi, Muhammad S. Zafar, Siraj D. Khan, Anhar Ullah, Ahmed Almatrafi, Khushnooda Ramzan, Sulman Basit

**Affiliations:** 1 Department of Pediatric Dentistry, College of Dentistry, Najran University, Najran, Saudi Arabia; 2 Center for Genetics and Inherited Diseases, Taibah University, Almadinah Almunawwarah, Saudi Arabia; 3 College of Dentistry, Taibah University, Almadinah Almunawwarah, Saudi Arabia; 4 Cardiac Sciences department, College of Medicine, King Saud University, Riyadh, Saudi Arabia; 5 College of Science, Taibah University, Almadinah Almunawwarah, Saudi Arabia; 6 Department of Genetics, Research Centre, King Faisal Specialist Hospital and Research Centre Riyadh, Riyadh, Saudi Arabia; 7 Department of Dental Materials, Islamic International Dental College, Riphah International University, Islamabad, 44000 Pakistan

**Keywords:** Clinical genetics, Dental diseases

## Abstract

Primary failure of tooth eruption (PFE) is a rare odontogenic defect and is characterized by failure of eruption of one or more permanent teeth. The aim of the study is to identify the genetic defect in a family with seven affected individuals segregating autosomal dominant non-syndromic PFE. Whole genome single-nucleotide polymorphism (SNP) genotyping was performed. SNP genotypes were analysed by DominantMapper and multiple shared haplotypes were detected on different chromosomes. Four individuals, including three affected, were exome sequenced. Variants were annotated and data were analysed while considering candidate chromosomal regions. Initial analysis of variants obtained by whole exome sequencing identified damaging variants in *C15orf40*, *EPB41L4A, TMEM232, KMT2C*, and *FBXW10* genes. Sanger sequencing of all family members confirmed segregation of splice acceptor site variant (c.1013-2 A > G) in the *KMT2C* gene with the phenotype. *KMT2C* is considered as a potential candidate gene based on segregation analysis, the absence of variant in the variation databases, the presence of variant in the shared identical by descent (IBD) region and *in silico* pathogenicity prediction. KMT2C is a histone methyltransferase and recently the role of another member of this family (KMT2D) has been implicated in tooth development. Moreover, protein structures of KMT2C and KMT2D are highly similar. In conclusion, we have identified that the *KMT2C* gene mutation causes familial non-syndromic PFE. These findings suggest the involvement of KMT2C in the physiological eruption of permanent teeth.

## Introduction

Tooth development and eruption is a complex process that is regulated by reciprocal interactions between epithelial and mesenchymal tissues at a cellular level^1^. A variety of signaling pathways and molecular players are associated with the initiation of tooth development, morphogenesis and tooth eruption. Wingless-type MMTV integration site family (Wnt), bone morphogenetic proteins (BMPs), fibroblast growth factors (FGFs), Sonic Hedgehog (Shh), parathyroid hormone (PTH) and ectodysplasin (Eda) pathways play a fundamental role during various stages of tooth development and eruption^1^. In addition, a number of transcription factors including Msx1, Msx2, Pax9, Lhx6, Lhx7, Dlx1, Dlx2, and Runx2 are necessary for early tooth morphogenesis^2^. Mutations in the component of above mentioned signaling pathways and transcription factors may lead to cessation of tooth eruption. Tooth eruption and development failure may be associated with a variety of pathological or idiopathic factors, mechanical interference, and interruption of the eruptive process^3,4^. Based on the observation that individuals experiencing a high frequency of hypodontia and having a family history for tooth eruption problems suggests a genetic involvement in the aetiology of eruption failure^5^.

Primary failure of tooth eruption (PFE) (MIM 125350) is a rare autosomal, non-syndromic disorder with complete cessation of the eruption of teeth and growth deficiency of the alveolar process in the affected region^5,6^. Eruption failure is defined as primary when the eruption process is arrested before the crown has penetrated the oral mucosa^7,8^. In PFE, posterior teeth (molars and premolars) are most commonly affected^9^. Heterozygous as well as homozygous mutations in parathyroid hormone receptor type 1 (*PTH1R*) have been identified as an underlying cause of PFE in several families^5,10–17^. However, the exact mechanism by which PTH1R-mutation leads to PFE is poorly understood^18^. It is noteworthy, that not all patients with PFE carry mutations in the *PTH1R* gene and the underlying genetics of PFE is unexplored.

The understanding of associated molecular and genetic mechanisms and the clinical management of tooth development and eruption disorders is quite challenging. This study was designed to identify the genetic defects underlying PFE. The identification of genetic factors associated with PFE will enable the clinician to better understand the aetiology, prompt diagnosis, carrier screening and clinical management of the affected individuals. Here, we have investigated a family with multiple affected individuals having PFE. Detailed clinical and molecular genetic analysis were performed and a potentially pathogenic mutation was identified in *KMT2C* gene as an underlying cause of PFE in this family.

## Methodology

### Ethical approval and Samples collection

**Figure 1:**
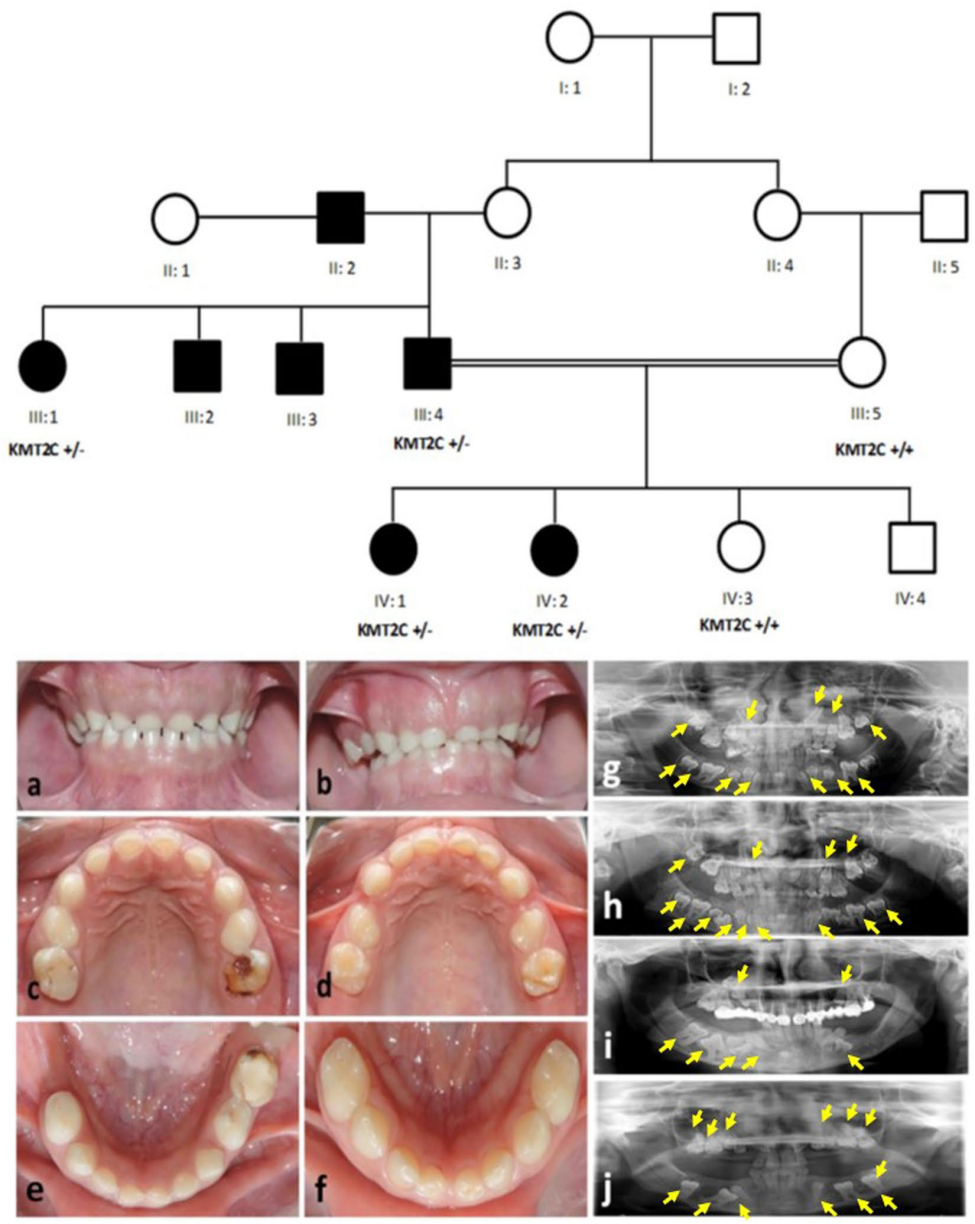
Pedigree of a four generations family segregating autosomal dominant PFE. Squares and circles represent males and females, respectively. Filled symbols indicate affected individuals. KMTC2 +/− indicate heterozygous individuals while KMTC2 +/+ show wild-type individuals. Clinical presentation of dentition of affected family members; (**a**) 14 years old affected female (IV: 1) and (**b**) 11 years old affected female (IV: 2), (**c,d**) labial view of primary dentition in centric occlusion showing generalized attrition and shortened crowns in individuals IV:1 and IV:2 respectively, (**e,f**) occlusal view of maxillary teeth showing functional primary teeth while there are no signs of eruption of permanent dentition or any pathology, (**e**) deciduous right lower second molar is missing in IV:1. (**g,h**) The radiographic (OPG) assessment of affected family members (IV: 1 and IV: 2) respectively, (**I,j**) their father and aunt (III:4 and III: 1). All OPGs confirmed the presence of multiple tooth buds embedded in the jaw bones (indicated using yellow arrows) that did not erupt in the functional occlusion. In addition, there were no mechanical or physical barriers suggesting the involvement of genetic components.

The scientific research ethics committee of the college of medicine, Taibah University approved the study protocols (051-02-2017). Written informed consents for genetic testing, clinical images and panoramic views (orthopantomograms) were obtained from all available subjects prior to participation. In case of minors (participants under the age of 18), informed consents were obtained from parents. All experimental procedures were carried out according to the Declaration of Helsinki. This study included a four generation family with seven individuals (4 males and 3 females) having primary failure of tooth eruption. Elder members of the family were interviewed and the pedigree was drawn (Fig. 1).

Two affected individuals (IV:1, IV:2) attended the speciality dental clinic (Almadinah, Saudi Arabia) presenting with the chief complaint of small teeth and were identified as having a primary failure of tooth eruption. The pedigree analysis showed an autosomal dominant inheritance of the tooth eruption phenotype.

Medical records of patients were reviewed in the detail and 3 ml peripheral blood samples were drawn for the present study from 6 available individuals (III: 1, III: 4, III: 5, IV:1, IV:2, IV:3) including 4 affected (III: 1, III: 4, IV:1, IV:2) and 2 normal (III: 5, IV:3) individuals (Fig. 1). DNA samples from these individuals were available for molecular analysis.

### Clinical examination of patients

A comprehensive radiographic and clinical evaluation was performed to determine a positive diagnosis of PFE. Any possible involvement of mechanical or secondary obstacles were ruled out. All participants were in good state of health. General physical examination and extra oral examination did not show any abnormalities. OPG, lateral cephalograph, blood biochemistry for calcium and phosphate levels were performed.

### DNA extraction and quantification

Whole blood samples were processed for genomic deoxyribonucleic acid (DNA) extraction using the ChargeSwitch® gDNA extraction kit (Thermofisher Scientific, 168 Third Avenue, Waltham, MA USA). The kit uses magnetic bead-based technology to isolate DNA from blood. The concentration and purity of DNA were determined using absorbance- and fluorescence-based quantification methods. For this purpose, MaestroNano spectrophotometer (Maestrogen, 8275 South Eastern, Las Vegas, USA) and Qubit 3.0 fluorometer (Thermo Fisher Scientific, 168 Third Avenue, Waltham, MA, United States) was used.

### Whole genome SNP genotyping

Single nucleotide polymorphism (SNP) markers were genotyped throughout the genome using DNA from six available individuals (III:1, III:4, III:5, IV:1, IV:2, IV:3). A detailed genotyping protocol has been described somewhere else^19^. A starting material of 200 ng DNA was used. Briefly, 200 ng DNA was dispensed in a deep-well plate followed by the addition of 4 µl resuspension buffer. In order to perform whole genome amplification, samples were denatured using 4 µl of 0.1 N NaOH and addition of 34 µl MA2 and 38 µl MSM solutions and incubation for 20–24 hours. The fragmentation master mix (FMS) was used to fragment samples. Fragments were precipitated and purified with 50 µl PM1 solution and 155 µl of 100% isopropanol, respectively. Samples were hybridized to bead chips using PB2 solution. Bead chips were stained and a single nucleotide extension was performed. PB1 solution was used to wash the bead chips in order to remove any incorporated nucleotides. Illumina iScan (Illumina, Inc., 5200 Illumina Way, San Diego, CA, USA) was used to image the bead chips. GenomeStudio software (Illumina Inc., 5200 Illumina Way, San Diego, CA, USA) was used for calculating logR ratio and B allele frequencies. For quality control, a positive and a negative control DNA sample was used in every chip. Subjects with overall genotyping efficiencies of at least 98% were selected. HomozygosityMapper, autoSNPa and dChip tools were used to detect shared homozygous regions^20,21^. DominantMapper was used to identify shared haplotype in all affected individuals^22^.

### Whole exome sequencing

Nextera rapid capture exome enrichment kit was used to sequence the complete coding region of the genome in three affected (III:1, III:4, IV:1) and one normal (IV: 3) individuals. This library preparation kit capture more than 214,000 exons (99.45% of the RefSeq genes). Briefly, tagmentation reagents in the Nextera exome kit were used to fragment and tag DNA followed by a PCR reaction to add sequencing adaptors and indices to fragments. Libraries were then denatured into a single stranded DNA followed by hybridization to biotin-labeled probes specific to the targeted region. Streptavidin beads were used to enrich the pool for the exonic regions only. Streptavidin bound biotinylated DNA fragments were collected from the solution using a magnetic stand followed by elution of the enriched DNA fragments. Enriched DNA fragments were amplified using primers complementary to sequencing adaptors. The targeted library is further loaded onto the flow cell for cluster generation and subsequent sequencing.

Paired end reads were obtained in the form of BCL files. These files were converted to fastq files by using the bcl2fastq algorithm. Illumina BaseSpace cloud was used to generate vcf files from fastq files. VariantStudio was used to annotate and filter the vcf files to identify common heterozygous variants in all affected individuals. AgileVCFMapper was used to identify any loss of heterozygosity (LOH) and common disease haplotype^23^.

### Gene enrichment analysis

Gene list enrichment analysis and candidate gene prioritization based on functional annotations and protein interactions network was used to identify the most relevant gene based on already known genes (training set) https://toppgene.cchmc.org/prioritization.jsp24 (Supplementary Table 1).

### Sanger Validation and Segregation analysis

Genotyping data revealed several candidate regions. Exome data analysis identified potentially damaging variants in at least five genes including *KMT2C*, *C15orf40, EPB41L4A, TMEM232* and *FBXW10*. The online version of Primer 3 software^25^ was used to design primers flanking candidate variants. Regions were amplified and sequenced using ABI 3500 Genetic Analyser (Applied Biosystems Inc. 850 Lincoln Centre Drive, Foster City, CA 94404 USA). All family members were screened for candidate variants in order to check the segregation of the variant with the disease phenotype.

### *In silico* analysis

Human splicing finder (http://www.umd.be/HSF3/credits.html) and NetGene2 (http://www.cbs.dtu.dk/services/NetGene2/) servers were used to predict the effect of splice donor site mutation (c.1013 – 2 A > G) in the *KMT2C* gene. Moreover, simple modular architecture research (SMART) tool (http://smart.embl-heidelberg.de/) was used to compare KMT2C protein domains with other members of the histone methyltransferases.

## Results

### Clinical description of cases

The intraoral examination of two affected females (IV:1, IV:2/age 11, 14) ages 14 years and 11 years showed retained deciduous teeth with shorter crown length (Fig. 1a,b). There were no evident signs of the eruption of permanent dentition. Both patients showed generalised attrition, healthy oral mucosa and soft tissues (Fig. 1a–f). All deciduous teeth were present in a healthy state; the only exception was missing deciduous right lower second molar extracted previously in individual IV:2 (Fig. 1e). Dental caries was present in deciduous upper second molars and left lower second molar in the same patient (Fig. 1e,f). Both cases were provisionally diagnosed with hypodontia. Clinical details of oral and dental features are presented in Table 1.

**Table 1:** Clinical presentation of oral and dental features of affected family members; IV:1 (14 years old female) and IV:2 (11 years old female).

Intra-oral examination	
Oral mucosa and soft tissues	Healthy
Deciduous dentition	• All deciduous teeth were present in healthy state (only right lower second molar was missing in IV:1) • Dental caries was present in certain second molars • Generalized attrition Shorter crown length
Permanent dentition	No signs of eruption in the oral cavity; all permanent teeth were missing
Radiographic examination	• Presence of developing tooth buds of permanent teeth (excluding third molars) • Presence of overlaying bone. Moreover, alveolar bone was also present in cases where primary teeth were extracted
Extra-oral examination	No obvious facial asymmetry or skeletal pathology was observed

In order to evaluate the status of un-erupted tooth buds, the radiographic analysis (OPG) was performed for affected and normal participants (Fig. 1g,h). The developing tooth buds of all permanent teeth (excluding third molars) were clearly observed in both affected patients (Fig. 1g,h). In addition, there was no mechanical obstruction hindering the path of eruption of permanent teeth. Therefore, the delayed tooth eruption had an unknown cause. Based on history, clinical and radiographic examination, the condition reflects a primary failure of tooth eruption (PFE) in which permanent tooth buds are present but failed to erupt in the oral cavity. Father (III:4) of affected individuals, paternal aunt (III:1) and uncles (III: 2 and III: 3) complained about multiple missing teeth while their respective OPG revealed the presence of several un-erupted tooth buds (Fig. 1I,j). A thorough clinical examination of the patients showed no extraoral skeletal features. The blood biochemistry results including calcium and phosphate levels were within the normal range. These finding confirmed the family history of non-syndromic PFE and possibility of genetic involvement, therefore, DNA samples from 6 family individuals were used for genetic analysis.

### Genotyping data analysis identified multiple regions shared by four affected individual

**Figure 2:**
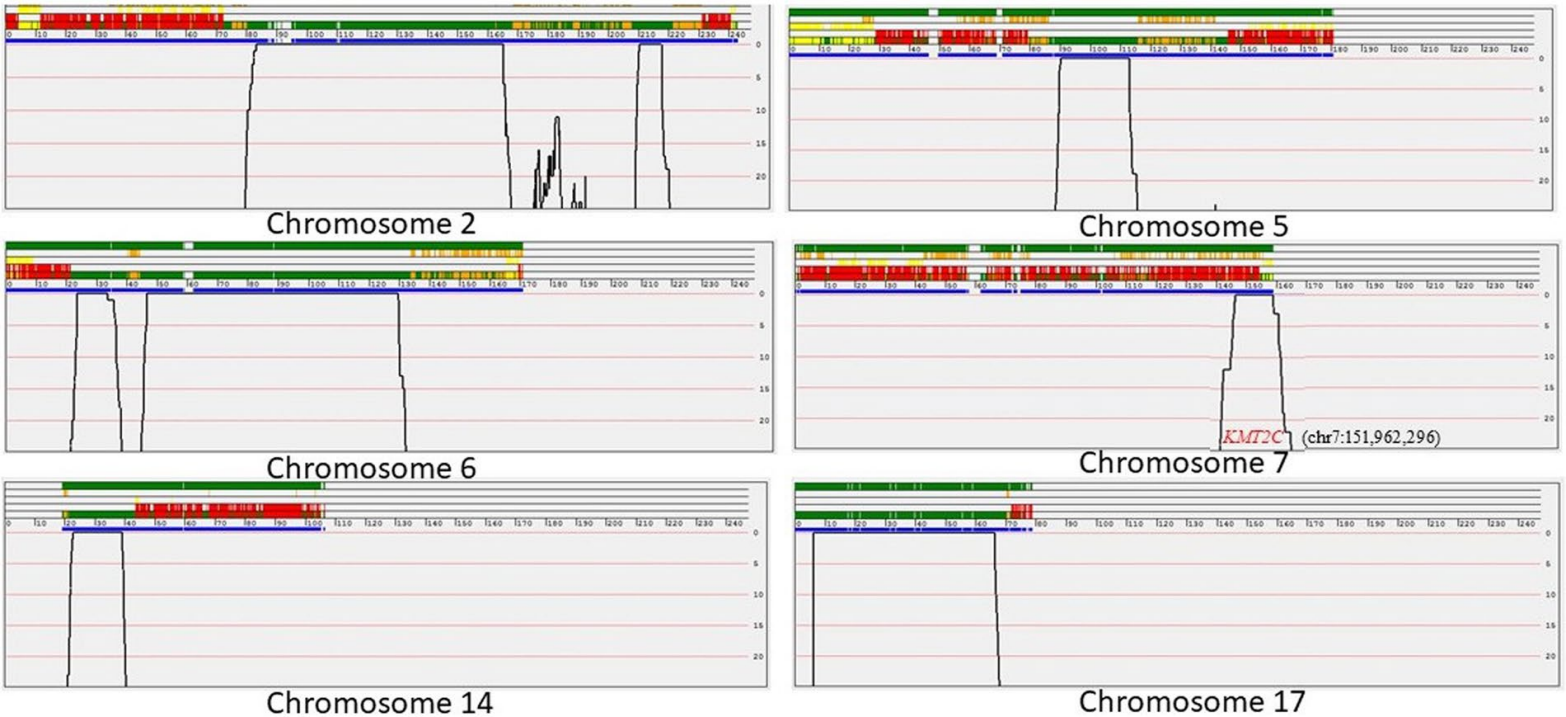
Graphical representation of DominantMapper output regions linked with the disease phenotype on different chromosomes. The result window is composed of two regions which display the analysis results. The upper region shows the results of the rule-based analysis for each SNP, while the lower region shows a graph of an empirically derived score, plotted against chromosome position. The chromosomal physical map position is shown between the two regions. The discontinuous thick blue line below the scale represents the positions of the SNPs, with gaps identifying regions with no SNP coverage. Green; SNPs that do not exclude linkage, Orange; SNPs that are excluded by affected relatives, Yellow; SNPs that are excluded by unaffected sibs, Red; SNPs that are excluded by affected sibs.

Genotypes of each SNP were determined using BRLMM clustering algorithm. Overall genotyping efficiencies of more than 98% were achieved for each sample. In addition, all samples were checked for gender matching and were found in agreement with individual sex. SNP genotypes were analysed using a variety of tools including HomozygosityMapper, AutoSNPa, dChip and DominantMapper. HomozygosityMapper, autoSNPa, and dChip failed to determine any shared region in all four affected individuals. This is in agreement with an apparently dominant mode of inheritance. An allele sharing analysis was performed using DominantMapper in order to identify at-risk haplotype(s). Multiple chromosomal regions were found where all affected pedigree members shared a chromosomal region identical by descent (IBD) (Fig. 2). Haplotype analysis showed that all affected subjects shared similar stretches of SNPs on chromosomes 2 (chr2:79836435-168306797), 5 (chr5:85812298-116016735), 6 (chr6:21213142-403287157), 7 (chr7:146462943-154764023), 14 (chr14:20378910-4370094715), 15 (chr15:27684347-608233664) and 17 (chr17:69854-70918976) (Fig. 2).

### Whole exome sequencing data analysis

Exome data of three affected (III:1, III:4, IV:1) and one normal (IV: 3) individuals were obtained from the NextSeq. 500 instrument with an average coverage of 80X. vcf files were uploaded to AgileVCFMapper software in order to identify homozygous stretche(s) shared by all affected. No common loss of heterozygosity (LOH) region was detected. Moreover, family based filters in the Illumina VariantStudio software did not identify any variant with pathogenic effect having a population frequency of less than 0.05. A variety of other filters were used including quality, frequency, genomic position, protein effect, pathogenicity and previous associations with the phenotype. Searching for disease causing variants present in homozygous or compound heterozygous state in all affected members and present in heterozygous or wild type state in the healthy individual of a family did not yield any candidate variant. Furthermore, exome data was searched for variants in known PFE genes and related family members. No pathogenic variant was obtained. Based on family pedigree, an autosomal dominant inheritance was considered. Only rare variants were taken into account (allele frequencies below or equal to 1% in 1000 G, ExAC (for exonic variants) and our in-house database), and only variants located within genes or promoter regions were considered. Initially, 16 genes were selected based on the presence of shared heterozygous variants (Table 2; candidate genes). Eleven genes were not considered for further analysis based on their irrelevance with the clinical phenotype (For example OR7G3 and OR8U8 are olfactory receptors and HERC2 variations are known to cause skin/hair pigmentation), high population frequency of the variants, low pathogenicity effect, and presence of these genes out of the shared stretches of haplotypes. This yielded a total of 5 unique heterozygous variants in *KMT2C* [c.1013-2 A > G; exon skipping], *C15orf40* [c.395dupT; p.Leu132fs], *EPB41L4A* [c.1933-2_1933-1insTTTTA; exon skipping], *TMEM232* [c.1819C > T; p.Arg607*] and FBXW10 [c.293_294delAG; p.Glu98fs] genes. These variants are present in the heterozygous state in all three affected individuals and absent in a healthy individual of a family. Segregation analysis using other affected and unaffected individual showed that variants in *TMEM232, KMT2C*, and *FBXW10* segregate with the disease phenotype while *C15orf40* and *EPB41L4A* variants failed to show segregation, therefore, *C15orf40* and *EPB41L4A* genes were ruled out. Further analysis revealed that variants in *FBXW10* and *TMEM232* genes are present with high frequency in Greater Middle East (GME) variome database (http://igm.ucsd.edu/gme/) and gnomAD browser (http://gnomad.broadinstitute.org/) (Table 3). A heterozygous splice acceptor site variant (c.1013-2 A > G) in the *KMT2C* gene is perfectly segregating with the disease phenotype and is not present in the polymorphism databases, and therefore, is considered as the only potential candidate variant. The variant identified in the *KMT2C* gene is predicted to cause exon skipping. Interestingly, the gene *KMT2C* is located on the shared haplotype on chromosome 7:146462943-154764023 region identified during whole genome genotyping data analysis (Fig. 2).

**Table 2:** List of training and test genes used for candidate gene ranking

Training genes	Test/Candidate genes
Msx2	PDE4DIP
Lhx6	TMEM232
Lhx7	PRIM2
Dlx1	SLC35G6
Dlx2	KRTAP9^−1^
Runx2	OR7G3
MSX1	TPTE
PAX9	ANKRD36
IRF6	ANKRD36
TP63	PRIM2
KMT2D	KMT2C
KDM6A	OR8U8
SATB2	PABPC3
TBX22	HERC2
TGFα	FBXW10
TGFβ3	SIRPB1
TGFβR1	SIRPB1
TGFβR2	
FGF8	
FGFR1	
KISS1R	
WNT3	
WNT5A	
CDH1	
CHD7	
AXIN2	
TWIST1	
BCOR	
OFD1	
PTCH1	
PITX2	
PVRL1	

### Gene enrichment analysis prioritized KMT2C as a top candidate gene

List of already known genes involved in tooth development and odontogenesis has been used as a training gene set. Genes that were considered as candidate genes during exome data analysis were used as test genes (Table 2). Both training and test genes were analysed using ToppGene suite^24^. *KMT2C* was ranked as a top candidate gene based on functional similarity to training gene list (Table 4).

**Table 3:** Population frequencies of variants on chromosome 5 (TMEM232) and 17 (FBXW10). Both variants are highly frequent in population. Data has been taken from gnomAD browser.

Gene	Variant	East Asian	South Asian	African	Jewish	European	Others	Total
TMEM232	Chr5:109756436 G > A	0.04268	0.02916	0.006213	0.005791	0.005631	0.02253	0.02199
FBXW10	Chr17;18647847AAG > A	0.000	0.00003249	0.02334	0.0004925	0.0004192	0.004489	0.002936

**Table 4:** ToppGene suite output. KMT2C gene was ranked as a top candidate gene based on training genes.

Rank	Gene Symbol	GO: Molecular Function		GO: Biological Process		Mouse Phenotype		Pubmed		Disease		Average Score	Overall pValue
		Score	pValue	Score	pValue	Score	pValue	Score	pValue	Score	pValue		
1	KMT2C	1.769E-1	1.321E-1	1.000E0	5.906E-2	9.880E-1	5.519e-2	1.000E0	1.336E-2	9.939E-1	3.079E-2	7.134E-1	8.355E-4
2	HERC2	4.730E-1	5.926E-2	9.998E-1	8.230E-2	9.960E-1	4.919E-2	3.666E-1	1.359E-1	1.000E0	1.026E-2	6.383E-1	5.217E-3
3	PRIM2	1.769E-1	1.321E-1	9.783E-1	1.251E-1			9.888E-1	3.486e-2	0.000E0	5.763E-1	3.879E-1	3.711E-2
4	PDE4DIP	7.322E-1	1.363E-1	2.427E-1	2.153E-1	1.939E-1	1.193E-1	1.575e-1	1.621e-1	3.425E-2	1.528E-1	2.291E-1	5.796E-2
5	PABPC3	1.769E-1	1.303E-1	1.392E-1	2.180E-1			0.000E0	5.809E-1			1.165E-1	1.794E-1
6	OR8U8	2.873E-1	9.043E-2	4.852E-1	1.977E-1			0.000E0	5.809E-1			1.968E-1	3.309E-1
7	OR7G3	2.688E-1	1.028E-1	4.852E-1	1.977E-1			0.000E0	5.809E-1			1.931E-1	3.444E-1
8	KRTAP9-1	0.000E0	6.048E-1	0.000E0	6.123E-1			0.000E0	5.809E-1			6.531E-3	4.114E-1
9	SIRPB1	2.490E-2	1.456E-1	8.599E-1	1.592E-1			0.000E0	5.809E-1	0.000E0	5.763E-1	1.587E-1	4.181E-1
10	TPTE	0.000E0	6.048E-1	5.392E-1	1.933E-1			8.994e-2	1.621e-1	0.000E0	5.763E-1	1.724E-1	4.571E-1
11	ANKRD36							0.000E0	5.809E-1	0.000E0	5.763E-1	0.000E0	5.786E-1
12	FBXW10	0.000E0	6.048E-1	0.000E0	6.123E-1			0.000E0	5.809E-1	6.229E-2	1.528E-1	4.160E-2	5.958E-1
13	TMEM232	0.000E0	6.048E-1	0.000E0	6.123E-1			0.000E0	5.809E-1			0.000E0	7.256E-1
14	C15orf40	0.000E0	6.048E-1	0.000E0	6.048E-1			0.000E0	5.809E-1			0.000E0	7.256E-1
15	SLC35G6	0.000E0	6.048E-1	0.000E0	6.123E-1			0.000E0	5.809E-1			0.000E0	7.256E-1

### Sanger sequencing of all family members confirmed segregation of KMT2C variant

**Figure 3:**
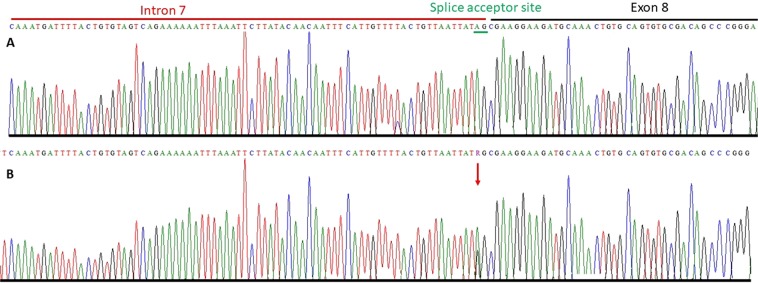
Partial sequence chromatogram of *KMT2C* gene. Upper panel shows a sequence of a normal individual from a family (**A**) while lower panel shows a nucleotides sequence of an affected individual (**B**). Arrow head indicates a mutation point.

Exon-intron boundaries and coding part of exon 8 of *KMT2C* gene was bi-directionally sequenced in all available family members including four affected (III:1, III:4, IV:1, IV:2) and two normal (III:5, IV:3) individuals (Fig. 1). *KMT2C* variant (c.1013-2 A > G) was found to be perfectly segregating with the disease phenotype. All affected individuals are heterozygous for the splice site variant while unaffected individuals are homozygous for the wild type allele (Fig. 3).

### *In silico* analysis revealed exon skipping

**Figure 4:**
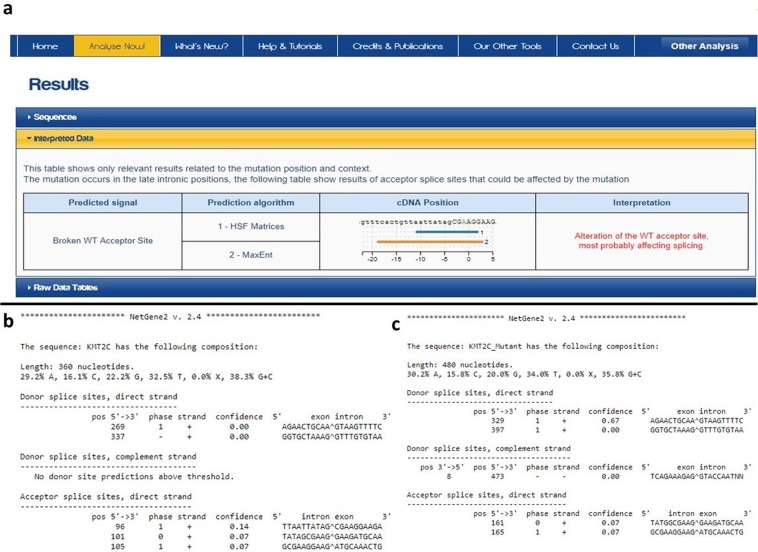
Analysis result of splice acceptor site variant using human splicing finder (**a**) and Netgene2 tools (b, c). Lower panel (**b**) shows 3 splice acceptor site in a wild type sequence while analysis of mutant sequence (**c**) shows 2 splice acceptor sites.

Human splice finder version 3.1^26^ and NetGene2 version 2.4^27,28^ predicted alteration of wildtype splice site as a result of splice acceptor site mutation (c.1013-2 A > G) resulting in exon skipping (Fig. 4). Furthermore, analysis of KMT2C and KMT2D protein sequences using simpler modular architecture research (SMART) tool^29,30^ determined that both proteins are structurally similar. Both KMT2C and KMT2D proteins have multiple plant homeodomains (PHD) at N-terminus, single high mobility group (HMG) domain and FY-rich (FYRN, FYRC) and SET domains at C-terminus.

## Discussion

In the current study, we have investigated a family having multiple individuals with PFE. In order to understand the underlying molecular and genetic mechanisms, clinical examination and a detailed molecular genetic analysis were performed for all available individuals. We have identified a splice site the mutation in the *KMT2C* gene as an underlying cause of PFE in this family. KMT2C is a member of histone methyltransferases (H3K4me3). Interestingly, a recent study has revealed the role of H3K4me3 in tooth development^31^, where authors have shown that methylation of the histones near the *WNT5A* gene plays an important role in dental development (odontogenesis). Actually, odontogenesis requires a two way interaction between dental epithelium and underlying mesenchyme. During odontogenesis, under the influence of several factors, the multipotent stem cells in the mesenchyme differentiates and become functional odontoblasts^32–34^. The role of Wnt5a as a regulator of odontogenic differentiation has been established^35^. The transcription activities of WNT5A are in turn epigenetically regulated by histone methyltransferases. Therefore, we hypothesize that defective histone modification of the WNT gene(s) by *KMT2C* product could affect tooth development in a similar way as mutations in the WNT gene might cause tooth abnormalities.

PFE is a rare non-syndromic disorder that arises as a result of odontogenic defects. No epidemiological studies have been performed to evaluate the prevalence of PFE^17^. Several genes have been identified that play a role in odontogenesis including *PAX9*, *MSX1*, *PTH1R*, and *AXIN2*^36–38^. Strong evidence exist that, in most of the cases, PFE is an autosomal dominant heterogeneous condition associated with mutations in *PTH1R* gene and the genes involved in activation of cAMP/PKA pathway in tooth eruption^39,40^. However, not all patients with PFE carry mutations in known genes and the underlying genetics of PFE is still unexplored^41^.

A presence of a family history of eruption failure along with the observation of multiple affected individuals experiencing a high frequency of hypodontia and tooth development problems suggests a genetic involvement in the aetiology of PFE^3,10,17^. In this study, we encountered a large family with an apparently autosomal dominant inheritance of PFE. Whole genome SNP genotyping followed by homozygosity mapping failed to detect any common loss of heterozygosity region. This ruled out the autosomal recessive inheritance of the PFE phenotype. SNP genotyping data were subjected to DominantMapper and multiple shared IBD regions were identified. Four individuals of a family were exome sequenced and a potentially pathogenic variant was identified in *KMT2C* [c.1013-2 A > G] gene. Segregation analysis confirmed the segregation of *KMT2C* [c.1013-2 A > G] with the disease phenotype in all available individuals of the family. Variant (c.1013-2 A > G) in *KMT2C* was considered as a potentially pathogenic variant based on its segregation with the PFE phenotype in the family, absence of *KMT2C* variant in genetic variation databases, known association of KMT2C family members with the tooth development and morphogenesis and presence of the gene in the IBD region on chromosome 7. Multiple *in silico* tools predicted that splice acceptor site mutation in *KMT2C* gene leads to exon 8 skipping. Exon 8 is important for the proper functioning of histone-lysine N-methyltransferase 2C as it encodes a zinger finger domain of the enzyme.

KMT2C and its other family members (KMT2A, KMT2B, and KMT2D) encode a SET domain containing lysine specific histone methyltransferases. These enzymes are responsible for tri-methylation of histone proteins (H3) at lysine 4 (H3K4me3). Lysine-specific histone methyltransferases perform a variety of functions. For instance, *KMT2A* knockdown significantly inhibit cell viability and cell migration and induce apoptosis while *KMT2B* mutations are associated with dystonia^42–44^. Mutations in *KMT2D* cause Kabuki syndrome^45^. Kabuki syndrome is an autosomal dominant disorder characterized by hypodontia, minor cleft lip with or without palate and craniofacial abnormalities^46,47^. We show that heterozygous splice site mutation in *KMT2C* likely cause autosomal dominant primary tooth eruption failure in humans. We consider *KMT2C* as a strong candidate gene for PFE phenotype based on the role of another member of histone methyltransferase (KMT2D) in tooth development and highly similar protein structure of KMT2C with KMT2D as determined by simpler modular architecture research tool (SMART).

In summary, we identified a new gene associated with PFE in a family with multiple affected individuals. Identification of genetic causes of isolated PFE can be used for the differential diagnosis of tooth developmental disorders. It helps in early diagnosis of family members of affected individuals and timely measures (such as patient education, proper care of primary dentition for prolonged functioning, and timely orthodontic evaluation) and may lead to appropriate treatment opportunities. As orthodontic treatment alone is not helpful in lately diagnosed PFE patients, therefore, timely genetic diagnosis of PFE can protect patients and clinicians from years of futile treatment and may benefit patients with more treatment choices.

## Supplementary information

Supplementary Table 1

## Data Availability

Genotyping data and vcf files are available on request.
