# Supplementary material for: KMT2C, a histone methyltransferase, is mutated in a family segregating non-syndromic primary failure of tooth eruption
Source: Sci Rep. 2019 Nov 11;9:16469. doi: 10.1038/s41598-019-52935-7 (PMC6848163; doi:10.1038/s41598-019-52935-7)

***KMT2C,* a histone methyltransferase, is mutated in a family segregating non-syndromic primary failure of tooth eruption**

Ali A Assiry, Alia M Albalawi, Muhammad S Zafar, Siraj D Khan, Anhar Ullah, Ahmed Almatrafi, Khushnooda Ramzan, Sulman Basit

**Supplementary Table 1:** Detail output of ToppGene suite analysis. KMT2C gene was ranked as a top candidate gene based on training genes.


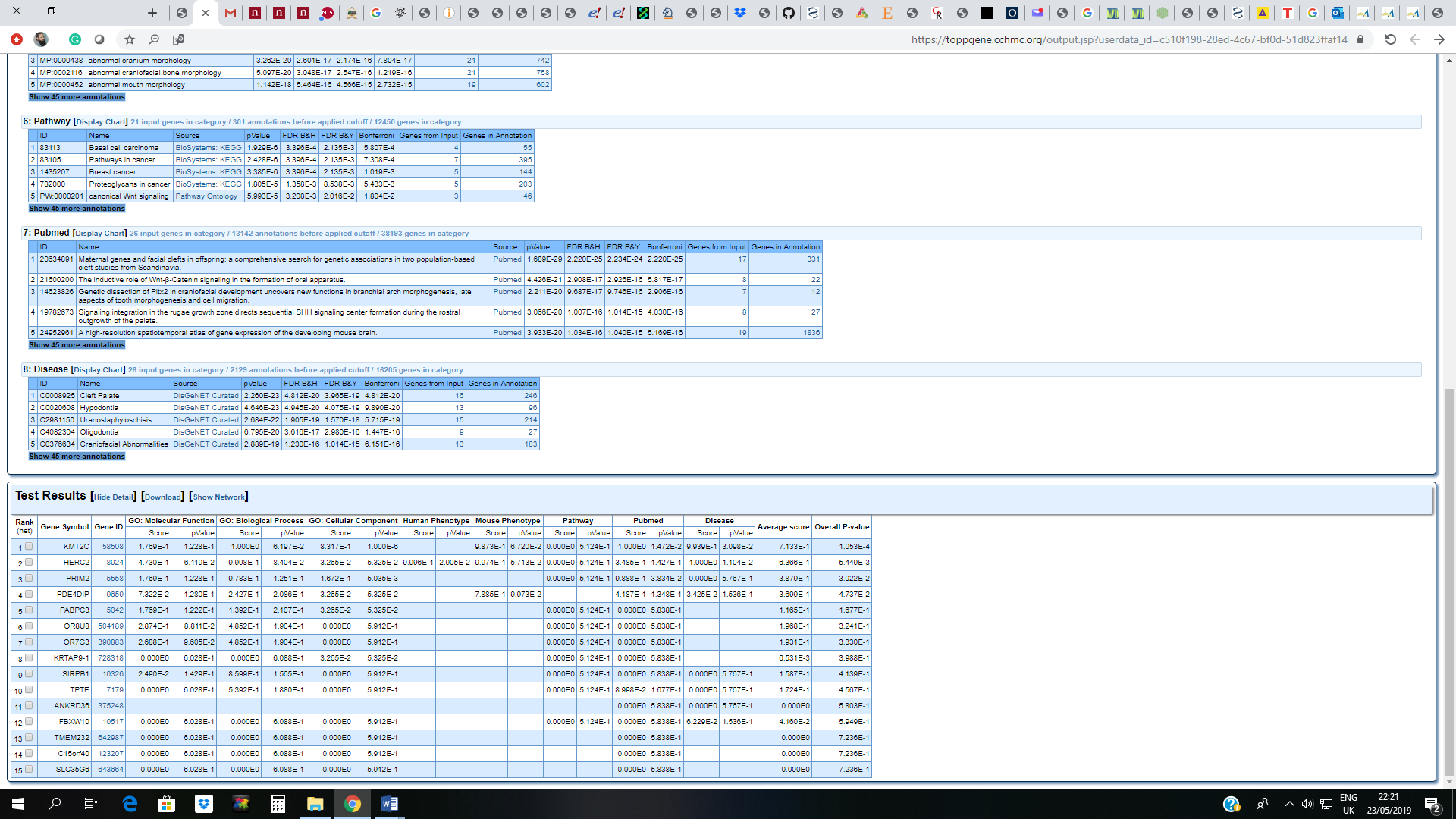

Supplement: Supplementary file 1 — Supplementary Table 1 [file 41598_2019_52935_MOESM1_ESM.docx]
